# The Role of the Notch Signaling Pathway in Recovery of Cardiac Function after Myocardial Infarction

**DOI:** 10.3390/ijms232012509

**Published:** 2022-10-19

**Authors:** Olga Kachanova, Arseniy Lobov, Anna Malashicheva

**Affiliations:** Laboratory of Regenerative Biomedicine, Institute of Cytology, Russian Academy of Sciences, 119991 Saint-Petersburg, Russia

**Keywords:** myocardial infarction, oxidative stress, fibrosis, hypertrophy, angiogenesis, cardiac regeneration, Notch signaling pathway

## Abstract

Myocardial infarction (MI) is a pathological process, evidencing as massive death of cardiomyocytes associated with hypoxic and oxidative stress. The formation of areas of fibrosis ultimately leads to heart failure. There are some mechanisms that contribute to the functional repair of the heart. In most mammals, including humans, the Notch signaling pathway has cardioprotective effects. It is involved in the formation of the heart in embryogenesis and in the restoration of cardiac function after MI due to: (1) reducing oxidative stress; (2) prevention of apoptosis; (3) regulation of inflammation; (4) containment of fibrosis and hypertrophy of cardiomyocytes; (5) tissue revascularization; and (6) regulation of proliferation and differentiation of cardiomyocytes. In addition, the Notch signaling pathway interacts with other signaling cascades involved in the pathogenesis of MI and subsequent cardiac repair. In this review, we consider the Notch signaling pathway as a potential target for therapeutic approaches aimed at improving cardiac recovery after MI.

## 1. Introduction

Myocardial infarction (MI) is a pathological process associated with the massive death of cardiomyocytes (CMC) due to ischemia. Cardiovascular disease accounts for 31% of the causes of death worldwide, claiming 17.9 million lives annually. Among them, 85% are associated with MI and stroke [1]. Worldwide, 32.4 million cases of MI are registered annually [2]. The recovery of the heart after MI is provided by a combination of signaling pathways and growth factors that stimulate the proliferation of certain cell populations (primarily CMC) [3]. Notch signaling plays a significant role in this [4]. Notch ensures cell-to-cell communication, regulates cellular differentiation, and interacts with other signaling pathways. This review considers the role of the Notch signaling pathway in the processes accompanying MI and the subsequent recovery of the heart muscle: apoptosis and CMC renewal, myocardial fibrosis, and angiogenesis. Understanding these mechanisms will help in the further development of effective methods for restoring myocardial function after MI. The Notch signaling pathway could be considered as a therapeutic target to mitigate the consequences of MI for the human body.

## 2. Pathogenesis of Myocardial Infarction

**Figure 1:**
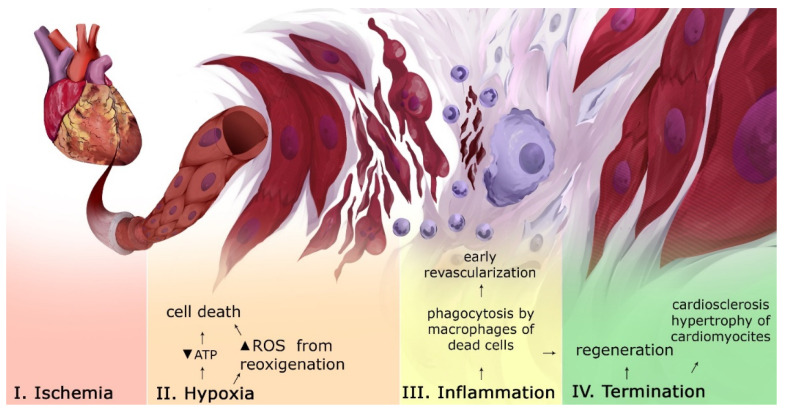
Tissue and cellular events in MI. After ischemic exposure, an increase in the level of ROS and the lack of ATP lead to cell death and a release of inflammatory mediators. During the inflammation stage, the following processes occur: phagocytosis of the dead cells’ debris and the formation of new capillaries. At the termination stage, some conditions for restoring the integrity of the myocardium by replacing dead CMC with fibroblasts and hypertrophy of surviving CMC occur. “Created with BioRender.com”.

The starting point of MI is ischemia of an area of the myocardium. Prolonged exposure to ischemia leads to necrosis of the heart muscle, which, in turn, leads to the accumulation of reactive oxygen species (ROS) (Figure 1) [5]. After restoring the blood flow, ROS could lead to ischemia-reperfusion complications, including the death of CMC [6]. Due to the destruction of CMC, a large number of molecules with a signaling activity are released into the intercellular space. At the inflammatory stage of MI, the infarct zone is cleaned of damaged cells and destroyed extracellular matrix (ECM). In case of a successful elimination of dead cell residues, inflammation is resolved and a reparative phase begins. It involves restoring the structural integrity of tissues by replacing dead CMCs with fibroblasts, hypertrophy of surviving CMC and the production of collagen produced by fibroblasts (scarring), and revascularization [7]. Due to insufficient regenerative capabilities of the heart, fibrous scars are formed, even if perfusion is restored. [8]. This increases the risk of complications due to (1) impaired conduction between CMC in the myocardium; (2) microvascular dysfunction; and (3) deposits of interstitial collagen, which can contribute to the stimulation of proteases that destroy the bonds between ECM and CMC [8]. The regulation of events in MI is based on many molecular interactions, including the Notch signaling pathway.

## 3. Notch Signaling Pathway

**Figure 2:**
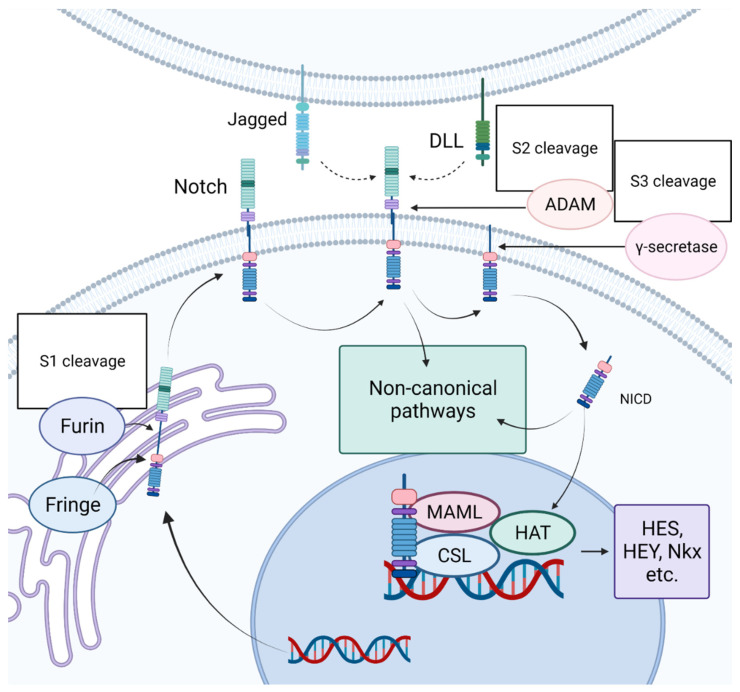
Schematic representation of Notch signaling pathway. A Notch ligand (DLL or JAG) binds to the Notch receptor (Notch1-4). The Notch intracellular domain (NICD) is cleaved when binding the receptor, and activated NICD enters the nucleus, where it interacts with intracellular regulatory complexes, after which the transcription of Notch target genes begins. “Created with BioRender.com”.

The Notch signaling pathway got its name from the gene originally identified in the laboratory of Thomas Hunt Morgan as being responsible for the appearance of notches on the wings of Drosophila melanogaster [9]. In the embryonic period, Notch plays an important role in development, determining the early formatting of the heart tube and the further maintenance of the heart [10]. In an adult organism, Notch acts as a regulator of cell proliferation and differentiation, controlling the maintenance of tissue identity [11,12,13,14]. At the same time, the biological effects of Notch largely depend on the cellular or tissue context [15,16]. A simplified representation of the Notch signaling pathway is outlined in Figure 2. The Notch receptor binds to a Delta-like (DLL) or Jagged (JAG) ligand. After that, the intracellular domain of the Notch receptor enters the nucleus, where it interacts with intracellular regulatory complexes, after which the transcription of target genes begins. Four Notch receptors (1–4) and five canonical ligands (JAG1-2, DLL 1, 3, 4) have been described in mammals. All of them are transmembrane proteins; therefore, direct intercellular interactions are necessary for the transmission of signals from the canonical Notch pathway [16]. The importance of the role of Notch signaling in the cardiovascular system has become clear not so long ago. Thus, in 2005, a mutation in the human NOTCH1 gene was described, and it became clear that mutations in the components of Notch signaling were associated with various cardiovascular pathologies [17]. Today, it is well accepted that Notch is an important component of the development and function of the cardiovascular system. The work of our group has shown that irregularities in the regulation of the Notch signaling pathway contribute to the pathogenesis of diseases such as calcification of the aortic valve, aneurysm of the ascending aorta, and myocardial infarction [18,19,20,21,22,23,24,25,26,27].

## 4. Role of the Notch Signaling Pathway in Myocardial Infarction 

### 4.1. Notch Reduces Oxidative Stress 

**Figure 3:**
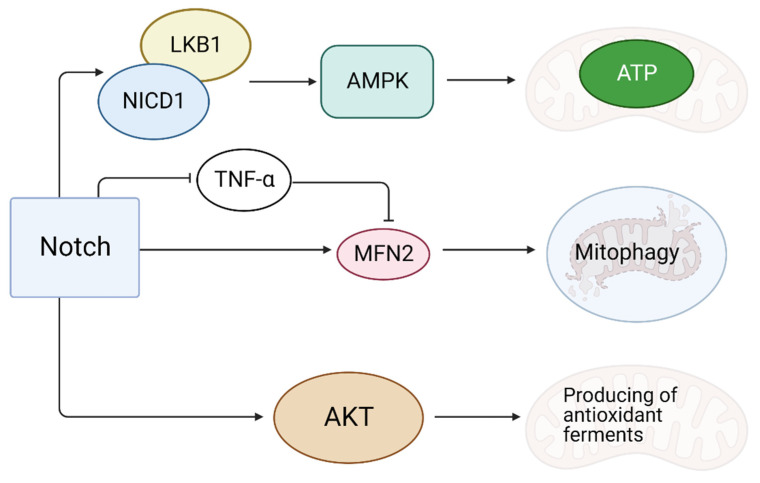
The role of the Notch signaling pathway in reducing oxidative stress at CMC. The launch of the Notch signaling pathway enhances the synthesis of the Mfn2 protein responsible for mitophagy. The Notch1 receptor can bind to the tumor necrosis factor-α and block its action. The production of antioxidant enzymes is controlled through the Akt cascade. Energy conservation in the cell is achieved by activating the AMPK cascade. “Created with BioRender.com”.

During reoxygenation after MI, the accumulation of ROS in the CMC is possible due to the continued production of energy [28]. ROS damage mitochondria and induce apoptosis, causing massive cell death [28]. It is assumed that the activation of Notch signaling enhances the synthesis of the Mfn2 (mitochondrial function-associated protein) protein responsible for mitophagy [29]. The Notch1 receptor could bind to tumor necrosis factor-α (TNF-α), a suppressor of Mfn2, thereby inhibiting mitophagy [29] (Figure 3). An anti-apoptotic effect of Mfn2 is partly due to the fact that it suppresses Drp1, which stimulates mitochondrial division [30]. The activation of Notch causes the Akt cascade activation, which is involved in the production of antioxidant enzymes in mitochondria [31]. In addition to this, NICD1 (the activated Notch1 intracellular domain) is able to bind to LKB1, which activates AMP-activated protein kinase (AMPK) at low energy levels in the cell [32]. AMPK activation promotes the reserve of cellular energy, accelerates the production and reduces the depletion of ATP, providing a cardioprotective effect after MI [32].

### 4.2. Notch Prevents Apoptosis of Cardiomyocytes

**Figure 4:**
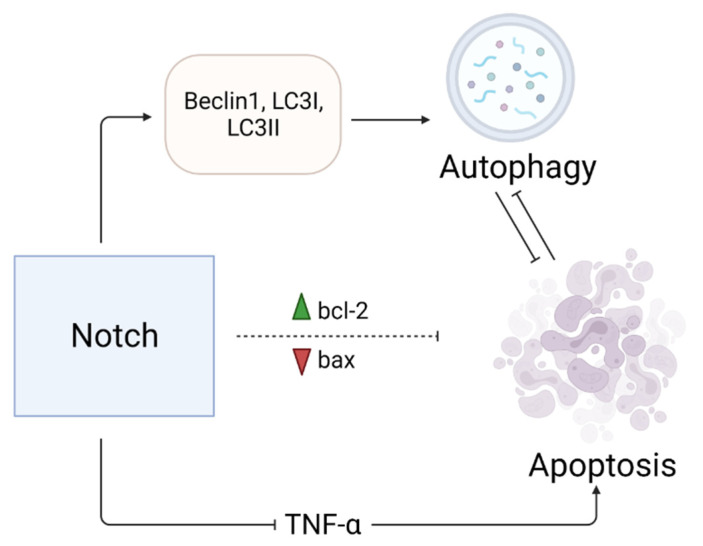
The significance of the Notch signaling pathway in preventing CMC apoptosis. Through the transmission of the Notch signal, a decrease in the expression of pro-apoptotic Bax and an increase in the expression of anti-apoptotic Bcl-2 is achieved. Additionally, the prevention of apoptosis can be achieved by increasing the expression of genes associated with autophagy. Inhibition of the pro-apoptotic tumor necrosis factor-α also helps prevent apoptosis. “Created with BioRender.com”.

The inactivation of the key Notch effector CSL (encoded by Rbpj gene) leads to a decrease in the amount of CMCs due to increased apoptosis resulting from an increase in the expression of proapoptotic Bax and a decrease in antiapoptotic bcl-2 levels [33]. It has been shown that the activation of Notch is associated with the expression of genes responsible for autophagy: Beclin1, LC3I and LC3II [34,35] (Figure 4). Notch1 is shown to have an anti-apoptotic role by shifting the balance towards autophagy [34,35].

### 4.3. Notch and Inflammation

**Figure 5:**
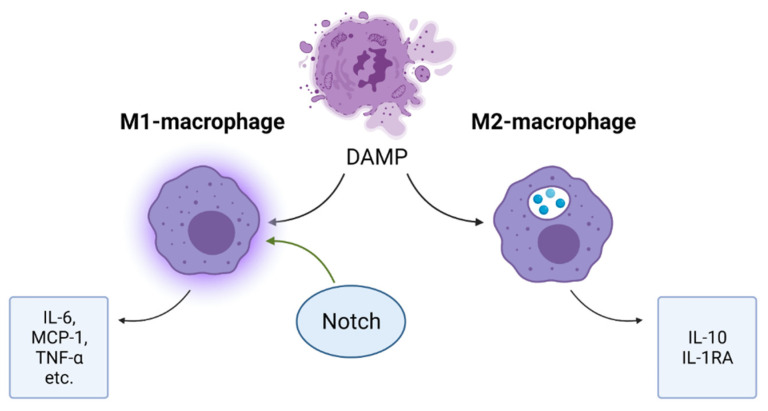
Effects of Notch pathway in inflammatory response. Notch signaling promotes the acquisition of a pro-inflammatory M1-macrophage phenotype that secretes inflammatory mediators.

Massive cell death in the affected cardiac area leads to an accumulation of inflammatory mediators and the activation of a cascade of inflammatory mediators, including inflammatory cytokines and chemokines. Recruited leukocytes enhance the production of inflammatory mediators and carry out phagocytosis of dying cells and tissue digestion by releasing proteases and oxidases [7]. An important role in inflammatory processes is played by macrophages, among which there are two types: M1 and M2 (Figure 5). M1-macrophages produce pro-inflammatory cytokines: interleukin-6, MCP-1 and TNF-α, etc.; M2 macrophages produce IL-10 and IL-1RA and other anti-inflammatory cytokines. In addition, M2-macrophages carry out phagocytosis of cell debris [36]. For the transition from the inflammatory phase to the reparative phase, the ratio of M2/M1 macrophages should increase. Similar results were achieved experimentally by blocking Notch in M1 macrophages. [37]. A pro-inflammatory role of Notch has been confirmed by the fact that macrophages induced by lipopolysaccharide, low-density lipoproteins, interferon-γ and IL-1 begin to express Notch ligand DLL4 on their surface. When DLL4 binds to a Notch receptor, macrophages begin to acquire a phenotype characteristic of the M1-type: the activation of the nuclear factor kB (NF-kB) pathway and the production of iNOS, TNF-α, IL-1β, IL-6 and IL-12 [38]. “Created with BioRender.com”.

### 4.4. Notch Signaling Pathway Reduces Myocardial Fibrosis

**Figure 6:**
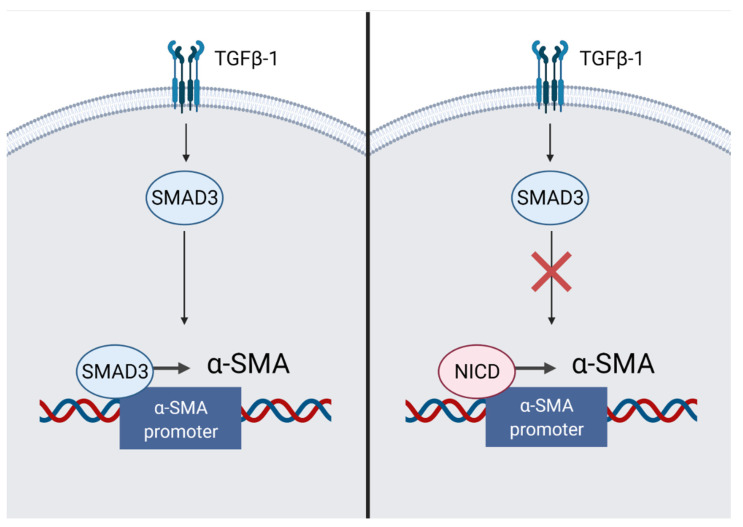
The mechanism of inhibition of myocardial remodeling in fibroblasts. The intracellular domain of the Notch receptor (NICD) inhibits signal transmission along the TGFß-1/SMAD3 pathway due to competition for the α-SMA promoter. “Created with BioRender.com”.

Myocardial fibrosis is an important aspect of maintaining the mechanical integrity of the heart. However, excessive fibrosis could lead to arrhythmias [39]. Cardiac fibroblasts maintain myocardial homeostasis by synthesizing collagen in sufficient quantities to keep the structural integrity of the heart. In case of serious damage, an increased load on the myocardium causes ventricular dilation. This triggers the renin-angiotensin-aldosterone system, causing an activation of angiotensin II. Activated angiotensin II increases tissue inflammation and the secretion of transforming growth factor-β (TGF-β), IL-1β and TNF-α. This contributes to an increased formation of myofibroblasts, an excess of which leads to fibrosis [40,41]. In addition to resident cardiac fibroblasts, CD45+ hematopoietic cells, vascular pericytes and immune cells could serve as sources of myofibroblasts [14]. Depending on the localization in the body, Notch is able to have various effects on fibrotic processes. In particular, in the neonatal myocardium, during the fibroblast-myofibroblast transition, the expression of the Notch1,3,4 receptor decreases [14]. In an adult heart, Notch inhibits TGF-β1/Smad3 signaling, thereby reducing fibrosis. NICD inhibits the TGF-induced transcription of α-SMA (Figure 6). It is possible that homeostasis is maintained due to the competition of NICD and Smad3 to bind to the promoter of the ACTA2 gene, encoding α-SMA. This balance may shift towards fibrosis when myofibroblasts are exposed to TGF-β1 [41]. However, the final role of Notch in the process of fibrosis remains ambiguous. In transgenic mice undergoing MI with an increased afterload, the induction of Notch using the immobilized DLL4 ligand promotes the generation of multipotent stromal cells with a myofibroblastic phenotype from epicardial cells of the population. These cells are able to differentiate into fibroblasts and cause reparative fibrosis [14,42].

### 4.5. Notch Negatively Affects Cardiomyocyte Hypertrophy

**Figure 7:**
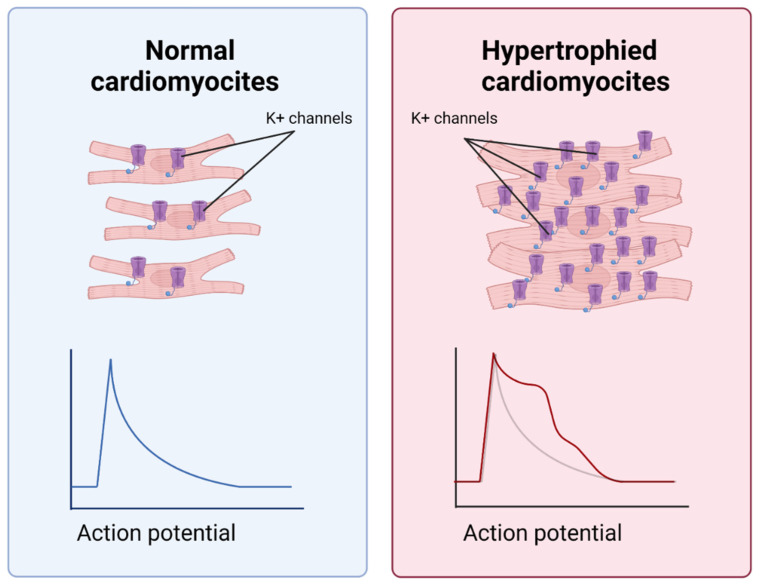
Differences between normal and hypertrophied CMCs. Hypertrophied CMCs differ from normal CMCs in terms of electrochemical features and a longer period of vulnerability to extra contractions. This is due to a change in the amount and conductive properties of potential-dependent potassium channels.

Massive death of CMC due to oxidative stress in hypoxia is a powerful signal for surrounding cells [5]. Dead cells form an empty space, causing mechanical stretching in the surviving CMCs and reducing their confluence. However, due to the limited proliferative potential, cardiomyocytes do not divide but undergo hypertrophy (Figure 7). Like fibrosis, hypertrophy is an adaptation that compensates for the massive death of myocardial cells. Nevertheless, its excessive level can cause further complications of MI. For example, in cardiac hypertrophy, arrhythmias may occur as a result of changes in the conductive properties and the number of potential-dependent potassium channels on the plasma membrane of cardiomyocytes. The window of vulnerability to extra contractions increases, and the probability of irregular contractions caused by premature stimuli increases [43]. Notch signaling may inhibit CMC hypertrophy [5,44]. Thus, it has been shown that the inhibition of the Notch1-Hes1 axis by a γ-secretase inhibitor (LY-411575) results in the increased expression of hypertrophic genes (ANP, BNP and β-MHC) [45]. “Created with BioRender.com”.

### 4.6. Notch Stimulates Angiogenesis

**Figure 8:**
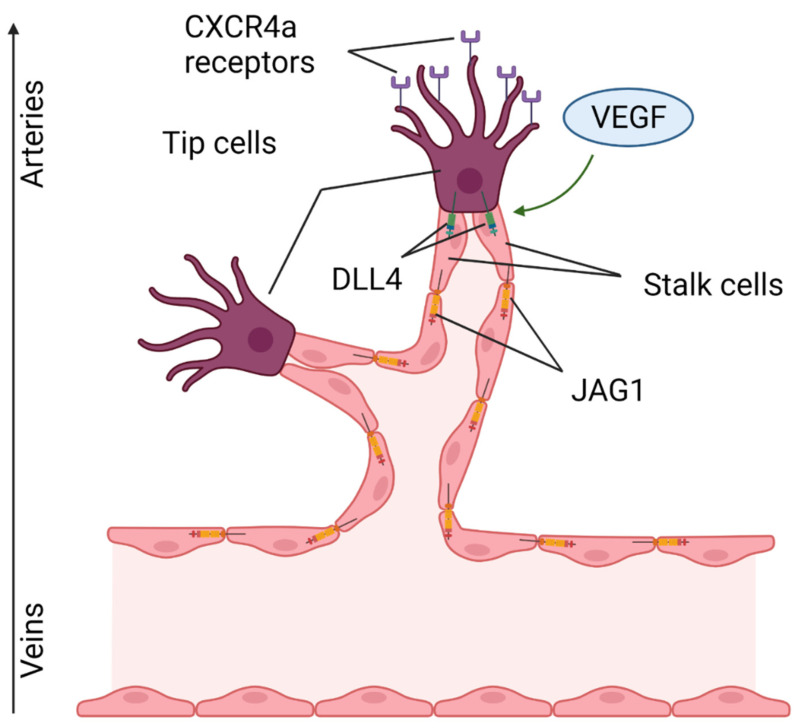
The role of Notch in angiogenesis. VEGF triggers the expression of DLL4 ligands in a leading tip cell, triggering the Notch in adjacent stalk cells. The direction of migration of tip cells is determined by the CXCR4 chemokine receptors. “Created with BioRender.com”.

A necessary requirement for the restoration of the damaged area after acute MI is the recovery of the capillary network in the ischemic area due to the formation of new blood vessels growing from existing ones [46]. Notch, along with vascular endothelial growth factor (VEGF), is an important regulator of angiogenesis (Figure 8). In a new-growing capillary, two types of cells are distinguished: tip (terminal, tip cells) and stalk (vessel wall cells). VEGF triggers the expression of DLL4 in a leading tip cell, which induces Notch signaling in the following stalk cells, resulting in an increase in the vessel diameter [47]. In this process, the Notch target is the chemokine receptor CXCR4a, which mediates endothelial migration. It is worth noting that tip cell migration proceeds in the venous-arterial direction. As soon as the cell approaches a great vessel, a decrease in the expression of CXCR4a is detected, which is important for preventing the excessive branching of blood vessels [48]. Tip and stalk cells may have different affinities to Notch ligands, due, for example, to the glycosylation of EGF repeats of Notch1 [49]. Due to these posttranslational modifications, the affinity of JAG1 ligand expressed in stem endotheliocytes is enhanced to the level of DLL4, and this ensures competition between them and prevents the excessive branching of the vascular network. This phenomenon is also provided by a higher level of expression of DLL1 ligand on the surface of stalk cells compared to DLL4 [15]. In conditions of ischemia, the expression of hypoxia-induced factor (HIF-1a) increases, and this could stimulate MSCs into secreting exosomes containing JAG1. When they enter endotheliocytes, they contribute to angiogenesis in the in vitro model of capillary tube formation. Similar results were obtained in vivo with the subcutaneous injection of JAG1-containing exosomes [50]. There are also contradictory data that indicate a decrease in the expression of receptors, ligands and target genes of Notch during ischemia/reperfusion [51]. In addition, a decrease in the expression of DLL4 leads to the formation of a more branched and rich vasculature [15].

### 4.7. Notch Regulates Proliferation and Differentiation

A contribution of resident stem cells to myocardial regeneration by their differentiation into mature CMCs is considered insignificant [52,53]. However, stem cell therapy still leads to a decrease in the size of infarction and a decrease in the proportion of hypertrophied CMCs [54]. We have recently obtained data on a more pronounced proliferative potential of the cardiac mesenchymal cells in the infarction zone [55]. Nevertheless, there are several examples (Danio rerio, axolotls, newborn baby rats, etc.) of regeneration of the vertebrate myocardium after injury [56,57,58]. In Danio rerio, this process is ensured by the proliferation of pre-existing (dedifferentiated) cells of the epicardium and endocardium [52,59]. During hypoxic damage, the cells of the epicardium and endocardium proliferate. Epicardial cells are capable of EMT, turning into myofibroblasts, which form a fibrous “framework” of the cardiac wall [60]. Notch stimulates the proliferation of undifferentiated cells and their migration to make contact with endocardial cells, which ensures the formation of a communication network between damaged and undamaged tissues. [61]. However, according to other data, the inhibition of Notch leads to an enhanced assembly of sarcomeric proteins typical of differentiated CMC [62]. Thus, Notch can hinder the differentiation of CMC [63]. It has also been shown that Notch disrupts the differentiation of fibroblasts into induced cardiomyocyte-like cells by blocking the transcription factor MEF2C (Myocyte Enhancer Factor 2C) independently of RBPJ-k [64]. In another study, the negative effect of the DLL4-Notch pathway on the expression of cardiomyocyte markers in MSCs (GATA4, cTnl) was also demonstrated [65]. There are different points of view about the role of Notch in the regeneration of the heart in mammals and Danio rerio. Thus, in Danio rerio, the Notch-mediated suppression of the expression of plasminogen activator inhibitor 1 (serpine1) in the proliferative phase (day 7 after ischemia) is observed in the endocardium, in contrast to mouse endocardial cells. The inhibition of serpine1 at an earlier time-point increased proliferation, which indicates its possible involvement in the regulation of myocardial regeneration [15]. There is evidence that after damage, the Notch1a, Notch1b, and Notch2 receptors are not expressed in CMC in Danio rerio [66]. This allows one to make an assumption about the paracrine action of the Notch pathway, whereby it exerts its effect with the help of extracellular vesicles produced by endocardial cells [59,67,68].

## 5. Conclusions

The Notch signaling pathway mediates cardioprotective effects at various stages of MI: ischemic damage to cardiomyocytes, at the reactive stage and during scarring. In the early stages of MI, Notch causes a decrease in oxidative stress by regulating the number of mitochondria and activating the Mfn protein. Notch also activates the Akt pathway, which, in turn, is involved in the production of antioxidant enzymes in mitochondria. Finally, Notch is involved in the activation of a pathway associated with AMP-activated protein kinase, which promotes energy reservation in the cell. The prevention of apoptosis is achieved by stimulating the production of survival factors (PI3K/AKT) and anti-apoptotic proteins. Thus, Notch stimulates the survival of the CMC, thereby reducing the damage from MI. Notch is able to reduce myocardial fibrosis by inhibiting the transmission of TGF-β1/Smad3 signals, reducing the production of ECM in myofibroblasts. However, it is worth considering the ability of Notch to activate the transition of epicardial cells into a population of multipotent stem cells with a myofibroblastic phenotype. Myocardial remodeling could also be prevented by Notch, because when Notch is inactivated, the expression of hypertrophy markers increases. During later stages after MI, the blood supply is restored, which is achieved by the growth of new vessels from existing ones. It is regulated by the combined action of VEGF and Notch. Notch also supports the homeostatic regulation of the endothelium. Despite many conservative features of the whole Notch signaling pathway, some aspects of its function differ in mammals and in Danio rerio. Due to epigenetic modifications, its role changes during the process of ontogenesis. The study of this issue is also complicated by the fact that the influence of Notch largely depends on the cellular and tissue contexts. This is partly due to the fact that the Notch signaling pathway is closely involved in cross-talk with other signaling pathways related to the systemic response to IM. HIF-1a, which is triggered under hypoxia, triggers Notch and, thereby, its target genes. Interactions between TNF-α and Notch give rise to various effects in different contexts. Nevertheless, from general observations one could assume a predominantly cardioprotective role of Notch in the pathogenesis of MI. This makes the development of methods for the modulation of the Notch signaling pathway, both during the early stages of MI and the long stage of subsequent recovery of the heart muscle, promising.

## Data Availability

Not applicable.
